# Differential Regulation of Microbial Community Succession by *Bacillus velezensis* and Its Impact on Flavor Compound Synthesis During Xiaoqu Baijiu Fermentation Stage

**DOI:** 10.3390/foods15203714

**Published:** 2026-10-19

**Authors:** Hong Ren, Yingying Xie, Shili Wu, Ren Huang, Qinghua Chen, Feng Chen, Jiajun Zhou, Zonghao Lan, Junheng Lv, Jianwei Zheng, Guidong Huang, Xianfeng Zhong

**Affiliations:** 1 College of Food Science and Engineering, Foshan University, Foshan 528231, China; helen231@126.com (H.R.); shareshare107@163.com (Y.X.); 18377437805@163.com (S.W.); hren2002@outlook.com (R.H.); 13117695067@163.com (Q.C.); 18877730373@163.com (F.C.); 15015611374@163.com (J.Z.); 19711325570@163.com (Z.L.); achero0518@163.com (J.L.); jwzheng@fosu.edu.cn (J.Z.); 2 Guangdong Traditional Fermented Food Engineering Technology Research Center, Foshan 528231, China; 3 Guangdong Province Food Circulation Safety Control Engineering Technology Research Center, Foshan 528231, China; 4 Foshan Brewing Engineering Technology Research Center, Foshan 528231, China; 5 Foshan Agricultural Biological Manufacturing Engineering Technology Research Center, Foshan 528231, China

**Keywords:** Xiaoqu Baijiu, *Bacillus velezensis*, bioaugmentation, microbial community, volatile flavor compounds

## Abstract

To address the demand for quality improvement in Xiaoqu Baijiu, this study evaluated bioaugmented fermentation using previously screened saccharification-promoting strains, *Bacillus velezensis* FUBL1 (B1) and FUBL3 (B3), to elucidate their impacts on physicochemical dynamics, microbial succession, and volatile flavor metabolism. High-throughput sequencing, Spearman correlation analysis, and flavor profiling were employed to track fermentation dynamics and cross-kingdom microbial interactions. The results demonstrated that bioaugmentation significantly enhanced sugar utilization efficiency. By the end of fermentation, total ester contents in fermented grains (*jiupei*) increased by 16.80% (B1) and 11.64% (B3) compared with the control. Upregulated long-chain fatty acid ethyl esters (e.g., ethyl laurate and ethyl palmitate) potentially contribute to enhancing floral/fruity aromatic qualities. From the perspective of microbial dynamics, *Bacillus* dominated the initial fermentation stage (up to 86.24%) and co-established a dominant niche with *Pediococcus* in mid-to-late stages. Exogenous inoculation accelerated the ecological dominance of *Saccharomyces cerevisiae* without competitive inhibition and increased the relative abundance of flavor-associated fungi like *Candida.* Spearman correlation analysis indicated that the succession of *Bacillus* was positively correlated with the enrichment of multiple core esters. Overall, this study demonstrates that targeted *Bacillus* bioaugmentation regulates microecological dynamics and flavor biosynthesis, providing a theoretical foundation for Xiaoqu Baijiu quality enhancement.

## 1. Introduction

Xiaoqu Baijiu is a highly representative traditional distilled spirit in China and across Asia, characterized by a unique flavor profile shaped by its semi-solid-state fermentation process [1]. This traditional brewing framework functions fundamentally as a semi-open, multi-species-driven fermentation system, where the ultimate liquor quality is fundamentally determined by structural composition and dynamic succession of the underlying microbial community. However, contemporary traditional Xiaoqu Baijiu production frequently encounters operational bottlenecks, including shortened fermentation cycles and overly simplified microbial metabolic networks. The constraints stem from an insufficient abundance of core flavor compounds, particularly key esters that dictate complex aromatic profiles. Consequently, the resulting product homogenization severely limits the high-quality development of the industry [2,3,4]. Targeted bioaugmentation through the strategic introduction of functional strains possessing high enzymatic or aroma-producing activities (e.g., *Bacillus* and *Saccharomyces*) has proven remarkably effective in elevating liquor quality and promoting the accumulation of key flavor metabolites [5,6,7]. Consequently, leveraging bioaugmentation strategies to reshape the fermentation micro-ecology represents a pivotal trajectory for overcoming flavor bottlenecks and enhancing overall product quality [8,9].

*Bacillus velezensis* has attracted widespread attention in brewing micro-ecological regulation due to its strong environmental adaptability and versatile extracellular enzyme systems (e.g., proteases, amylases, and cellulases), which provide significant advantages in degrading macromolecular substrates and generating flavor precursors [10,11]. In our previous work, two *B. velezensis* strains with marked saccharification-promoting capacity, designated FUBL1 and FUBL3, were successfully isolated from a traditional Xiaoqu Baijiu brewing system. These strains effectively boosted glucoamylase/amylase activities, promote reducing sugar generation, and stimulate the release of signature flavor compounds during the saccharification stage of a simulated brewing system. However, saccharification represents merely the initial phase of Xiaoqu Baijiu production; the subsequent main fermentation stage is the critical period for determining ultimate flavor quality, involving complex microbial successions and metabolic network reconstructions [12]. Crucially, superior performance by exogenous strains during saccharification does not guarantee their ecological adaptation or sustained functionality in the subsequent fermentation stage, nor do metabolic advantages gain early on transfer seamlessly to the later phase. Prior studies focused primarily on the saccharification step, leaving a gap in our systematic understanding regarding the colonization stability of these two strains during subsequent fermentation, their capacity to reshape microbial community structures, and their downstream impacts on volatile flavor synthesis.

Accordingly, this study investigated these saccharification-promoting *B. velezensis* strains—FUBL1 and FUBL3 (hereafter referred to as B1 and B3)—by comprehensively evaluating the dynamic variations in physicochemical metrics, microbial community succession, and volatile metabolites throughout the fermentation process. Emphasis was placed on examining the bio-perturbations triggered by these exogenous strains, alongside constructing correlation networks between key microbial taxa and signature flavor components. Ultimately, this work aims to elucidate the ecological functions and underlying mechanisms of saccharification-promoting *B. velezensis* during the main fermentation phase, thereby providing a theoretical foundation for micro-ecological modulation and quality improvement in high-grade Xiaoqu Baijiu brewing.

## 2. Materials and Methods

### 2.1. Strains and Materials

*B. velezensis* B1 and B3 were previously isolated from Xiaoqu Baijiu fermented mash in our laboratory. Glutinous rice was purchased from Heilongjiang Heliang Agricultural Co., Ltd. (Harbin, China). Jiuqu (fermentation starter) was obtained from Hunan Yongzhou Yada Technology Industrial Co., Ltd. (Yongzhou, China). All other chemical reagents used in this study were of analytical grade.

### 2.2. Preparation of Bioaugmented Inoculum

*B. velezensis* strains B1 and B3 were separately inoculated into Luria-Bertani (LB) liquid medium and cultured at 37 °C with shaking at 160 rpm for 12 h. Subsequently, a 2% (*v*/*v*) inoculum was transferred into fresh LB liquid medium and cultivated under the same conditions until the cell concentration reached 10^7^ CFU/mL. The resulting bacterial cultures were utilized as the bioaugmented inocula for subsequent fermentation.

### 2.3. Fermentation Process and Sampling of Xiaoqu Baijiu

The Xiaoqu Baijiu fermentation was conducted according to the procedure reported by Hu et al. [13] with minor modifications adapted to laboratory conditions (Figure S1).

The brewing process comprised glutinous rice soaking, steaming, starter (Jiuqu) inoculation, saccharification, and subsequent fermentation. Briefly, 2.5 kg of glutinous rice was soaked in tap water (raw material-to-water ratio of 1:0.7, *w*/*w*) for 1 h and steamed for 1 h. Upon cooling to 35 °C, the steamed rice was thoroughly mixed with 0.6% (*w*/*w*, relative to rice weight) Jiuqu and 15% bacterial inoculum (*v*/*w*, cultured to 10^7^ CFU/mL) [14]. For the control group (CK), an equivalent mass of Jiuqu and an equal volume of sterile medium were added. The mixtures were loaded into 15-L fermenters equipped with one-way air-release valves and incubated at 30 °C for 24 h to allow aerobic saccharification. Following saccharification, tap water was added at 1.5 times the original rice weight (*w*/*w*), and the mixture was subjected to anaerobic fermentation at 30 °C for 15 days. For each treatment (CK, B1, and B3), one independent fermentation vessel was used. Fermented grain (*jiupei*) samples were collected periodically during fermentation, with three parallel samples of approximately 50 g collected from the same fermentation vessel at each time point for subsequent analyses. Portions of the collected samples were stored at −20 °C for physicochemical characterization, while the remaining aliquots were stored at −80 °C for volatile metabolite analysis via gas chromatography–mass spectrometry (GC-MS) and high-throughput sequencing.

### 2.4. Determination of Physicochemical Properties and Flavor Compounds in jiupei

#### 2.4.1. Determination of Physicochemical Properties of *jiupei*

Reducing sugar concentration was determined using the 3,5-dinitrosalicylic acid (DNS) colorimetric method. Total soluble solids were measured directly using a handheld refractometer. The pH and total titratable acidity were determined according to the methods described by Wan et al. [15].

#### 2.4.2. Determination of Volatile Flavor Compounds in *jiupei*

Volatile organic compounds (VOCs) in *jiupei* samples were analyzed using an Agilent headspace solid-phase microextraction combined with gas chromatography–mass spectrometry (HS-SPME–GC–MS) system. Briefly, 200 mg (approximately 200 μL) of *jiupei* sample was weighed into a 20-mL headspace vial, followed by the addition of 10 μL of internal standard solution (2-octanol, 10 mg/mL). In a separate headspace vial, 10 μL of an *n*-alkane mixed standard solution (C_9_–C_23_, 10 mg/L) was added to determine retention indices (RIs). SPME extraction was performed using a 50/30 μm DVB/CAR/PDMS fiber (StableFlex, 2 cm). Gas chromatographic separation was conducted on a DB-Wax capillary column (30 m × 250 μm × 0.25 μm). High-purity helium was used as the carrier gas at a constant flow rate of 1.0 mL/min in splitless mode, with a septum purge flow rate of 3.0 mL/min. Samples were preheated at 60 °C for 15 min, extracted for 30 min, and desorbed at the injection port for 4 min. The oven temperature program was initiated at 40 °C (held for 4 min), ramped to 245 °C at 5 °C/min, and held at 245 °C for 5 min. Mass spectrometry was operated in electron impact (EI) ionization mode at 70 eV. The temperatures of the injection port, transfer line, ion source, and quadrupole were maintained at 250 °C, 250 °C, 230 °C, and 150 °C, respectively. Full-scan data were acquired over a mass-to-charge ratio (m/z) range of 20–400 with a solvent delay of 2.37 min. Volatile organic compounds were identified by searching mass spectra against the National Institute of Standards and Technology library (NIST 23, National Bureau of Standards, Gaithersburg, MD, USA) [16]. Qualitative identification was confirmed by combining spectral similarity scores with experimentally determined retention indices (RIs), calculated using a homologous series of *n*-alkanes under identical chromatographic conditions. The mass spectral similarity matching threshold was set between 200 and 1000. Semi-quantitative determination of target compounds was performed using the internal standard method according to Equation:

$$C=\frac{C_{2}\times V_{2}}{V}\times \frac{A_{1}}{A_{2}}$$

where “*C*” represents the relative concentration of the target compound (mg/L); *A*_1_ is the peak area of the target compound; *A*_2_ is the peak area of the internal standard; *C*_2_ is the mass concentration of the internal standard (mg/mL); *V*_2_ is the volume of the internal standard (mL); and *V* is the sample volume (L).

To evaluate the sensory contribution of individual volatile components, aroma activity values (OAVs) were calculated using Equation :

$$\mathrm{OAV}=\frac{\mathrm{Ci}}{\mathrm{OTi}}\times 1000$$

where “*Ci*” is the relative concentration of compound (mg/L), and *OTi* is the odor threshold of compound *i* (µg/L, listed in Table S2).

### 2.5. High-Throughput Sequencing of Microbial Communities

Total genomic DNA of the microbial community in *jiupei* was extracted using the E.Z.N.A.^®^ Soil DNA Kit (Omega Bio-tek, Norcross, GA, USA). The V3–V4 hypervariable region of the bacterial *16S rRNA gene* was amplified using universal primers 338F (5′-ACTCCTACGGGAGGCAGCAG-3′) and 806R (5′-GGACTACHVGGGTWTCTAAT-3′). The fungal *ITS1* region was amplified using the primers ITS1F (5′-CTTGGTCATTTAGAGGAAGTAA-3′) and ITS2R (5′-GCTGCGTTCTTCATCGATGC-3′). The quality of the PCR products was examined via 1% agarose gel electrophoresis, and DNA concentrations and purities were quantified using a NanoDrop 2000 spectrophotometer (Thermo Scientific, Wilmington, DE, USA). Sequencing libraries were constructed using the NEXTFLEX Rapid DNA-Seq Kit and subsequently sequenced on the Illumina MiSeq PE300 platform (Illumina, San Diego, CA, USA). Raw sequencing reads were quality-filtered using fastp and assembled using FLASH. Denoising and amplicon sequence variant (ASV) generation were conducted using the DADA2 plugin (version 2020.2) within QIIME2 (version 2020.2) under default parameters. Taxonomic classification of the resulting ASVs was performed using the Naive Bayes classifier in QIIME2, against the Silva database (Release 138) for bacteria and the UNITE database (Release 8.3) for fungi. The raw sequencing datasets have been deposited in the National Microbiology Data Center (NMDC) database under project number NMDC40130265~NMDC40130309 (16S), NMDC40130310~NMDC40130354 (ITS).

### 2.6. Statistical Analysis

Group differences in alpha diversity metrics (Chao1 index and Shannon index) were evaluated using the Kruskal–Wallis test. Upon detecting significant differences, Dunn’s post hoc pairwise multiple comparison test with Bonferroni correction was applied. Principal coordinate analysis (PCoA) and non-metric multidimensional scaling (NMDS) were performed based on Bray–Curtis dissimilarity matrices to assess microbial community structure. Statistical differences in beta diversity across groups were evaluated using analysis of similarities (ANOSIM) with 999 permutations. The ANOSIM R-statistic ranges from −1 to 1, where an R-value approaching 1 indicates that inter-group variation exceeds intra-group variation; *p* < 0.05 was considered statistically significant. All microbial community bioinformatic analyses and visualizations were conducted using the Majorbio Cloud Platform (https://cloud.majorbio.com).

Physicochemical data were compiled using Microsoft Excel 2021 and are expressed as mean ± standard deviation (SD). Bivariate correlation analyses between key microbial taxa and volatile flavor compounds were performed in IBM SPSS Statistics 26. Two-tailed P-values were calculated for each taxon–metabolite pair. To control the false discovery rate (FDR), *p*-values across the correlation matrices were adjusted using the Benjamini–Hochberg (BH) procedure. Graphical plots and heatmaps were generated using Origin 2024.

## 3. Results

### 3.1. Dynamic Changes in Physicochemical Properties and Volatile Flavor Compounds During Xiaoqu Baijiu Fermentation

To systematically track the dynamic evolution of the micro-environment and metabolic products throughout the fermentation process, physicochemical properties of *jiupei* were continuously monitored at high frequency across the entire fermentation cycle (days 0, 1, 2, 3, 5, 10, and 15). Meanwhile, based on the stage-specific characteristics of flavor compound synthesis and accumulation, steady-state time points during the mid-to-late fermentation stages (days 5, 10, and 15) were selected for volatile flavor profile analysis.

#### 3.1.1. Changes in Sugar Content and Acidity

**Figure 1:**
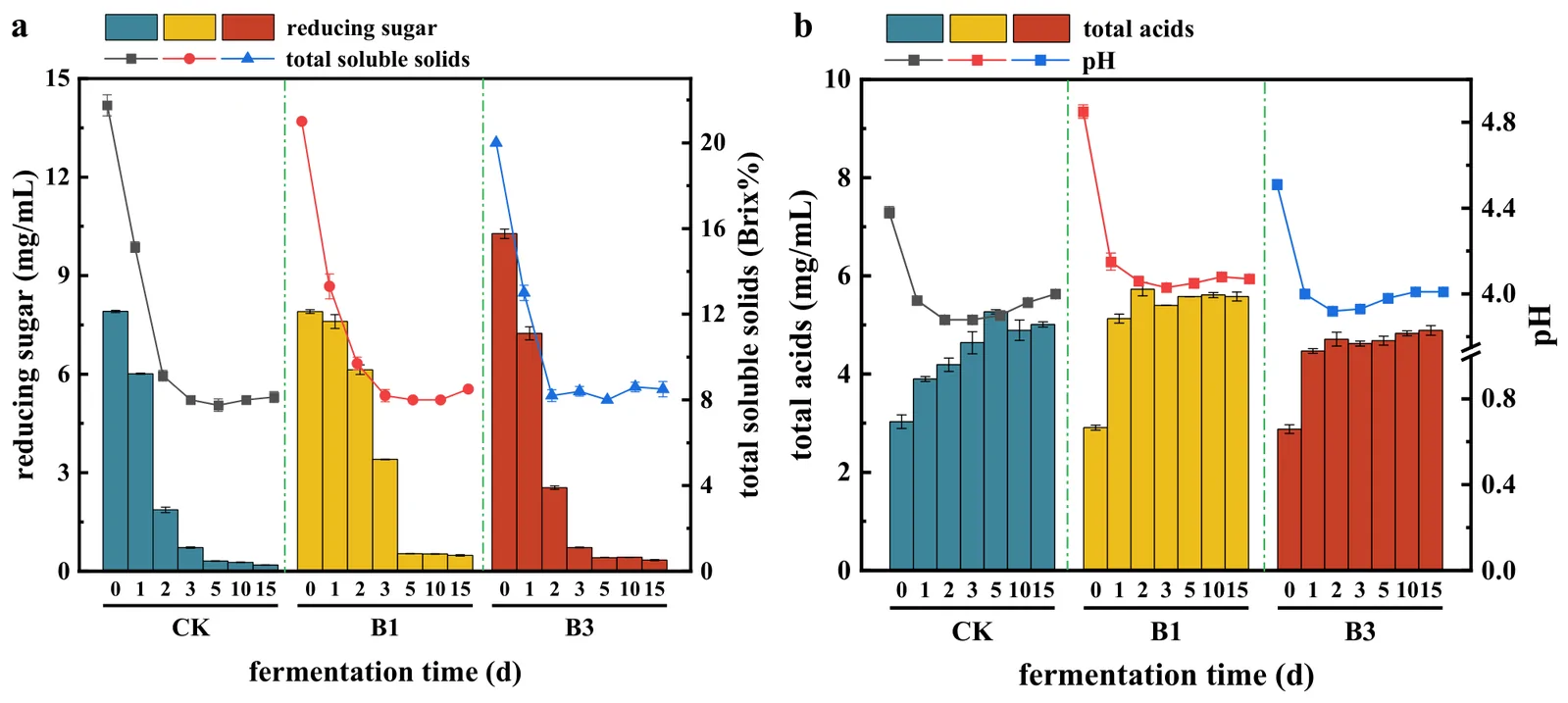
(**a**) Changes in sugar content during *jiupei* fermentation; (**b**) changes in acidity during *jiupei* fermentation.

As shown in Figure 1a, total soluble solids levels across all three groups ranged from 20% to 22% at the beginning of fermentation (day 0), then declined rapidly to approximately 8% as starch hydrolysis proceeded before stabilizing. This indicated that macromolecular carbohydrates directly accessible to microorganisms were largely depleted. Reducing sugars peaked on day 0 (7.9–10.3 mg/mL) and subsequently dropped sharply due to consumption by microorganisms such as yeasts, leveling off below 0.5 mg/mL by day 5. Notably, the B3 group exhibited a more pronounced decrease in reducing sugars, demonstrating that bioaugmentation effectively enhanced sugar utilization efficiency [17].

As depicted in Figure 1b, total acidity in all three groups displayed an initial rapid increase followed by stabilization as fermentation progressed. During the first 3 days, total acidity accumulated rapidly within the system, which was likely attributable to the rapid proliferation of acid-producing microorganisms such as lactic acid bacteria during the early stage. In the mid-to-late stages, total acidity gradually reached an equilibrium, mainly due to nutrient depletion and the participation of free organic acids in esterification reactions. The initial total acidity across all groups ranged from 2.88 to 3.03 mg/mL. During the first 3 days, total acidity levels in both the B1 and B3 groups were higher than those in the CK group, with the B1 group exhibiting the greatest overall increase. This suggests that the introduction of *B. velezensis* reshaped the microbial community structure and metabolic functions within the fermentation system, thereby promoting the synthesis and accumulation of organic acids. Concurrently, the pH dropped rapidly during the early stage and stabilized between 3.88 and 4.88 during the mid-to-late stages, indicating that the addition of exogenous strains caused no significant perturbation to the overall pH trajectory. This acidity regulation trend aligns with the findings reported by Huang et al. [18] regarding *Bacillus licheniformis*-bioaugmented Baijiu fermentation.

#### 3.1.2. Analysis of Volatile Flavor Compounds in *jiupei*

**Figure 2:**
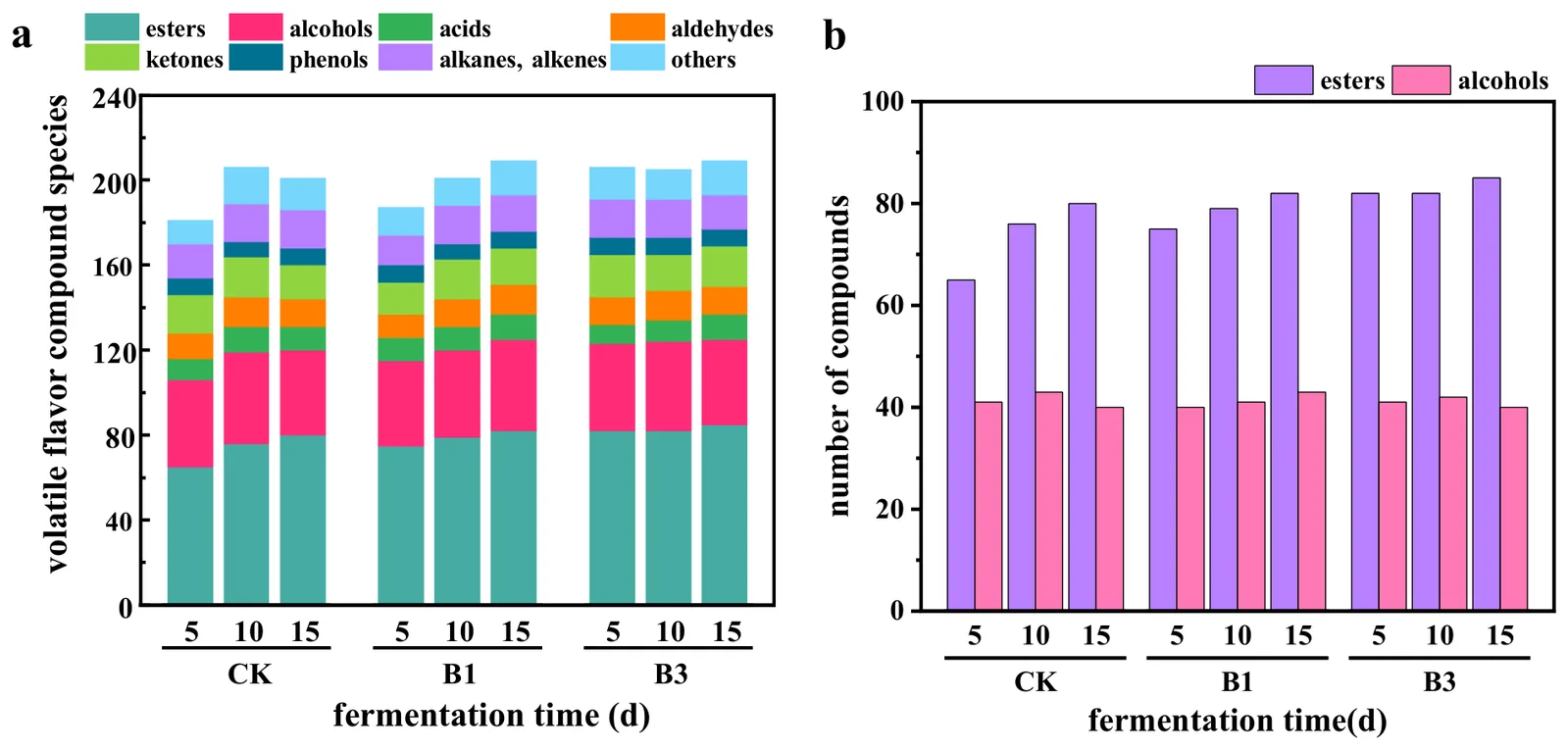
Changes in volatile flavor compounds’ species in *jiupei* during fermentation: (**a**) all compounds; (**b**) esters and alcohols (the number of other low abundance compound species is shown in Figure S3).

Volatile organic compounds (VOCs) constitute the chemical foundation of the distinct aroma and taste of Xiaoqu Baijiu, with their concentrations and proportions directly dictating overall liquor quality. In this study, a total of 221 VOCs were identified across the three groups of *jiupei* samples, including 87 esters, 43 alcohols, 12 acids, 15 aldehydes, 21 ketones, 8 phenols, 18 alkanes and alkenes, and 17 other compounds. As illustrated in Figure S3, esters and alcohols were the predominant VOC classes, with total compound diversity exhibiting an upward trajectory throughout fermentation. As depicted in Figure 2, volatile compound diversity in the control group (CK) expanded from 180 species on day 5 to 200 on day 15. The bioaugmented groups (B1 and B3) exhibited similar upward trajectories, displaying marked increases in total compound counts from day 5 to day 15. Overall, both B1 and B3 maintained slightly higher numbers of volatile species than the CK group across fermentation stages (Table S1). Regarding chemical composition, esters remained the most abundant class throughout fermentation, maintaining a dominant proportion at all time points, followed by alcohols. Additionally, organic acids, aldehydes, ketones, phenols, alkanes/alkenes, and other minor classes collectively contributed to the complex volatilome network. Dynamic variations in the relative proportions of these chemical classes across fermentation stages and treatment groups highlight the regulatory role of bioaugmentation in shaping the volatile flavor profile.

**Figure 3:**
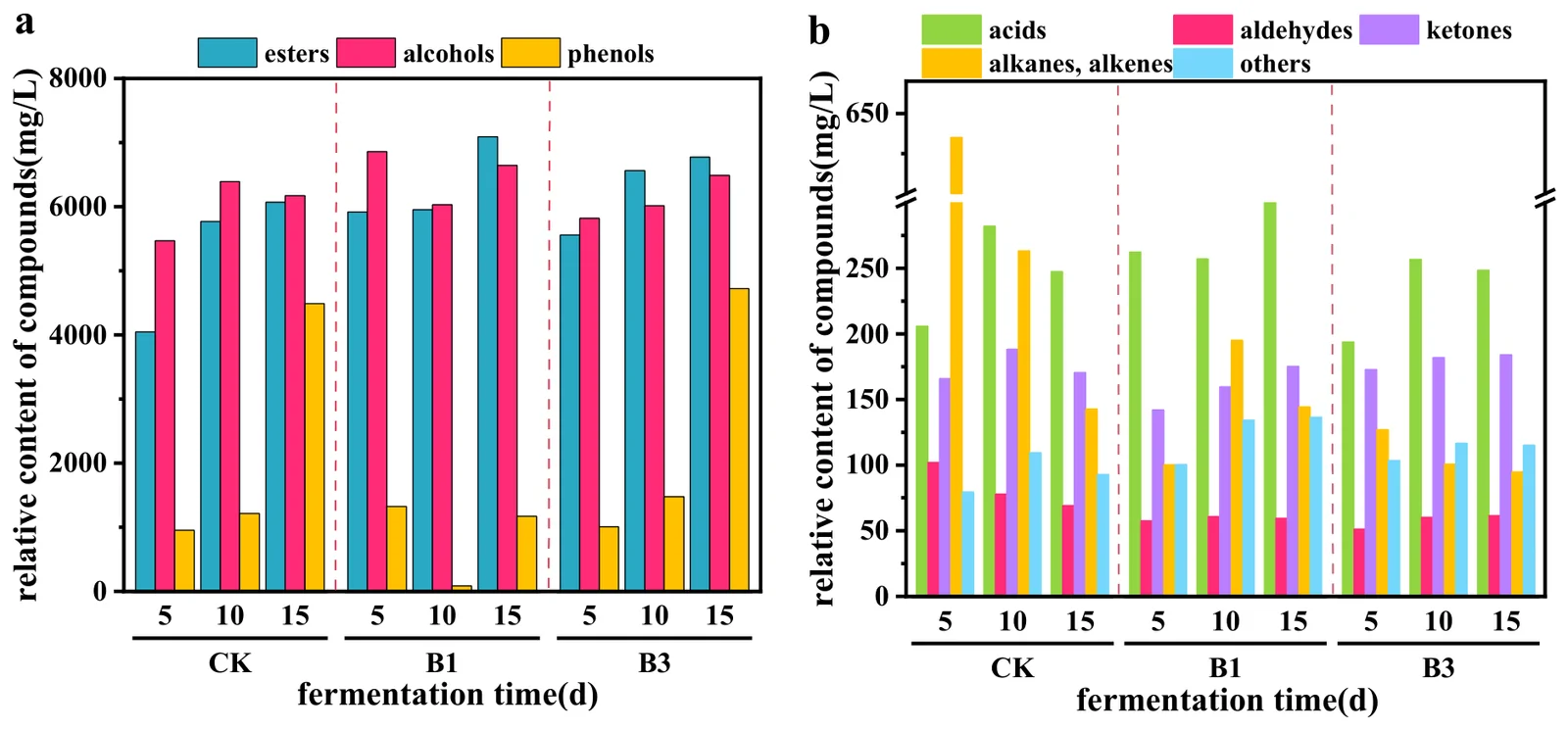
Changes in volatile flavor compounds’ content in *jiupei* during fermentation: (**a**) esters, alcohols, phenols; (**b**) acids, aldehydes, ketones, alkanes and alkenes, others.

Figure 3 depicts the relative concentration shifts in volatile flavor compounds across treatment groups throughout fermentation. Among all volatile classes, esters and alcohols remained consistently predominant, serving as the core constituents of the primary flavor profile of Xiaoqu Baijiu. By the end of fermentation (day 15), total ester concentrations in the B1 and B3 groups reached 7087.49 and 6774.70 mg/L, representing increases of 16.80% and 11.64%, respectively, relative to the CK group (6068.05 mg/L). Aroma activity value (OAV) analysis (Table S2, Supplementary Materials [19,20,21,22,23,24,25,26,27]) revealed that multiple ester compounds exhibited OAVs > 1, confirming them as key contributors to the characteristic fruity and floral attributes of the spirit. Notably, bioaugmentation with strains B1 and B3 upregulated the accumulation of long-chain fatty acid ethyl esters (LCFAEEs), including ethyl laurate, ethyl arachidate, and ethyl myristate. Previous work by Wu et al. [28] highlighted that LCFAEEs play a pivotal role in mitigating spirit pungency while enhancing overall “smoothness” and “roundness” on the palate. Taken together, these results demonstrate that bioaugmentation with *B. velezensis* altered the volatile profile of *jiupei*, which may have implications for the aroma characteristics of the final spirit; however, such effects require validation after distillation and sensory evaluation.

At the end of fermentation, alcohol concentrations in the B1 and B3 groups were also higher than those in the CK group. As vital aroma-enhancing components in Baijiu, higher alcohols serve as key precursor substrates for ester formation, and their presence at appropriate levels imparts body and richness to the liquor [29]. The B1 and B3 groups exhibited enhanced alcohol accumulation capacity during the late fermentation stage, which was likely associated with the efficient carbohydrate metabolism of *B. velezensis* [30] and the amino acid degradation pathway in yeasts [31], thereby providing a sufficient precursor supply for ester synthesis. The dynamic trajectory of phenolic compounds in the B3 group mirrored that of the CK group, reaching comparable levels by day 15. In contrast, the phenolic content in the B1 group on day 15 was markedly lower than that in both the CK and B3 groups. This reduction suggests that the introduction of strain B1 exerted a distinct biological perturbation on the indigenous *jiupei* microbiome, suppressing the abundance or metabolic activity of specific native taxa responsible for phenolic metabolism. Although present at much lower levels than esters and alcohols, volatile classes such as acids, aldehydes, and ketones exhibited distinct dynamic shifts across stages and treatments. For instance, aldehyde concentrations in the CK group decreased from 102.05 mg/L on day 5 to 69.15 mg/L on day 15, whereas the B1 and B3 groups maintained lower, stable levels ranging from 51.16 to 61.67 mg/L (Figure S3 and Table S1, Supplementary Materials). This trend likely reflects the metabolic reduction of aldehydes to corresponding alcohols or their consumption in secondary condensation reactions.

In summary, bioaugmentation with exogenous *B. velezensis* significantly enhanced both the diversity and concentrations of volatile flavor compounds (particularly esters and alcohols) in *jiupei*, which may contribute to enriched flavor complexity. Regarding compound diversity, both bioaugmented groups harbored higher numbers of volatile compound species than the CK group, with B3 group maintaining the highest diversity throughout. In terms of total concentration, the B3 group exhibited a distinct advantage by the end of fermentation (day 15), underscoring its superior efficacy in driving flavor metabolite synthesis. Conversely, the B1 group generally remained close to the CK group with minor stage-specific fluctuations, highlighting metabolic regulatory capacities between different bacterial strains.

#### 3.1.3. Comparative Analysis of Volatile Metabolites Between Bioaugmented and Control Groups

**Figure 4:**
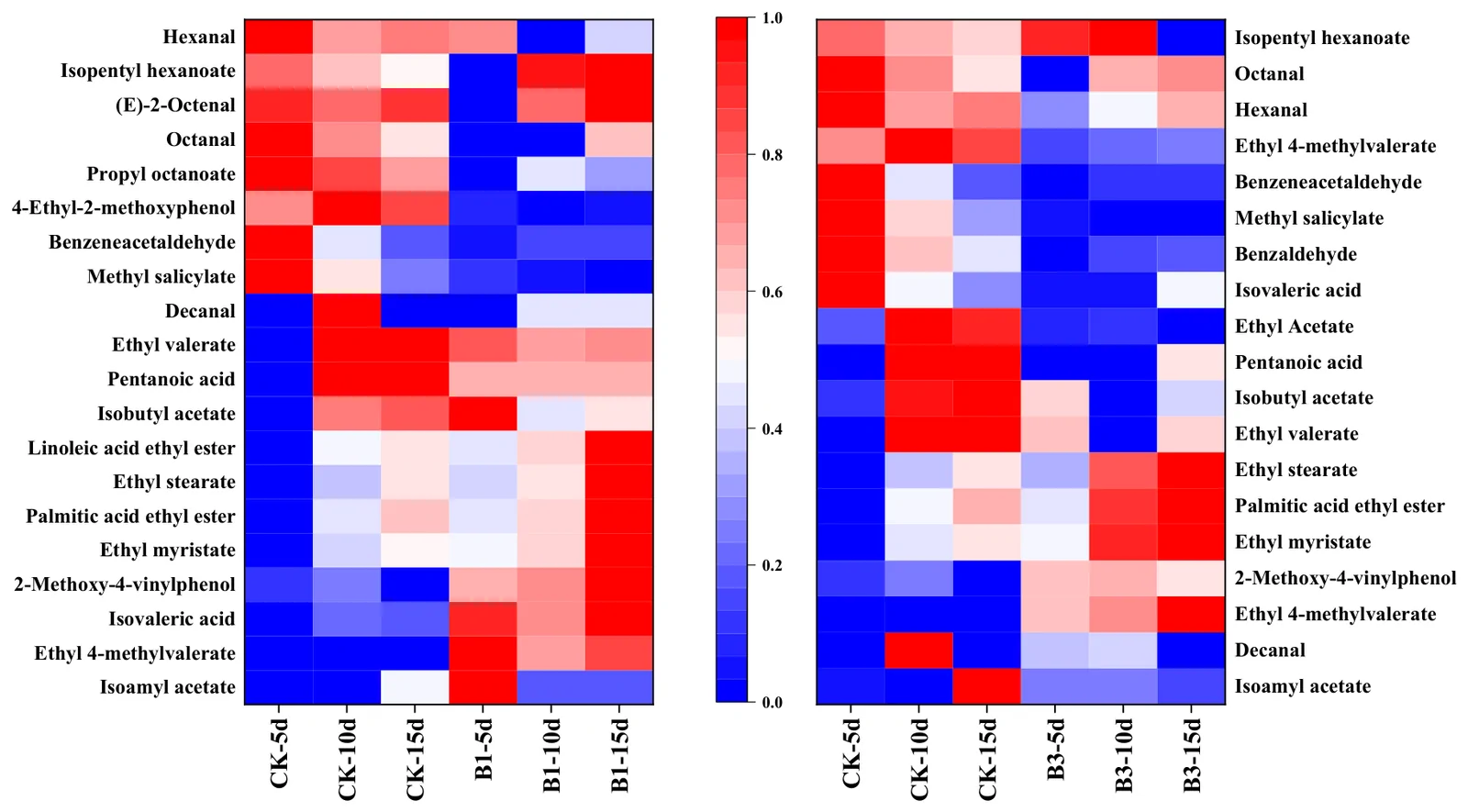
Difference in volatile flavor compounds between bioaugmentation fermentation and traditional fermentation.

To further elucidate the specific impacts of *B. velezensis* bioaugmentation on the volatile profile of Xiaoqu Baijiu, differential volatile compounds between the bioaugmented groups (B1, B3) and the CK group were screened from the 221 identified volatile constituents. The screening criteria were set to |log_2_FC| ≥ 1 and OAV > 1. Heatmaps were subsequently generated based on min–max normalized data (Figure 4).

As illustrated in Figure 4, 20 differential volatile flavor compounds were identified between the B1 and CK groups (11 esters, 5 aldehydes, 2 phenols, and 2 acids), while 19 differential volatiles were identified between the B3 and CK groups (10 esters, 5 aldehydes, 2 acids, and 2 phenols). In the CK group, hexanal (imparting grassy and raw green off-notes) and pentanoic acid (a short-chain fatty acid conferring sour and pungent odors) accumulated at elevated levels throughout fermentation. In contrast, bioaugmentation with B1 and B3 suppressed these undesirable flavor compounds to minimal levels across the entire fermentation span (days 5–15). This suppression suggests that *B. velezensis* inoculation may be associated with reduced off-flavor accumulation, potentially through metabolic transformations or altered interspecific microbial competition, thereby potentially contributing to greater flavor purity of the fermented mash. Esters represent essential aroma-active compounds in Xiaoqu Baijiu. LCFAEEs such as ethyl palmitate, ethyl stearate, and ethyl myristate impart body, richness, and depth to Baijiu [32]. While the accumulation of these LCFAEEs remained low in the CK group throughout fermentation, these compounds were significantly upregulated during the middle-to-late stages in both the B1 and B3 groups. Furthermore, sweet and fruity esters, including isopentyl hexanoate and linoleic acid ethyl ester, reached peak concentrations in the B1 group at the end of fermentation. Additionally, 2-methoxy-4-vinylphenol, a critical contributor to the smoky aroma character in Baijiu [33], exhibited more stable and sustained accumulation in the B1 and B3 groups during the mid-to-late stages compared with the CK group.

Distinct strain-level flavor metabolic signatures were observed between *B. velezensis* B1 and B3. Strain B1 exhibited a pattern of continuous flavor accumulation throughout the entire process, with core flavor compounds (e.g., ethyl laurate and related LCFAEEs) reaching their maximum concentrations at the termination of fermentation (day 15), which may potentially influence the aroma complexity and fullness of the liquor. In contrast, strain B3 displayed robust aroma-producing activity during early-to-mid fermentation, with key fruity esters synthesizing rapidly and peaking around day 10 before undergoing a minor decline toward the final stage. This phenomenon suggests that the rapid early flavor-producing potential of strain B3 holds practical promise for shortening or optimizing fermentation schedules; however, its ultimate impact on distilled spirit quality warrants further confirmation via professional sensory evaluation.

### 3.2. Effect of B. velezensis Bioaugmentation on Microbial Community Succession During Xiaoqu Baijiu Fermentation

#### 3.2.1. High-Throughput Sequencing Data and Alpha Diversity

To trace the succession dynamics of microbial communities throughout the fermentation process, *jiupei* samples were collected at five time points (days 0, 3, 5, 10, and 15) from both the CK group and bioaugmented groups (B1 and B3) for *16S rRNA gene* and *ITS1* high-throughput sequencing. The sequencing yielded 2,400,554 and 2,120,646 high-quality clean reads for bacteria and fungi, respectively, with average read lengths of 429 bp and 413 bp. Following ASV denoising and taxonomic annotation, 726 bacterial ASVs and 172 fungal ASVs were identified. The Good’s coverage for every sample exceeded 99.0%, indicating that the sequencing depth was sufficient to capture the vast majority of microbial species within the system.

The Alpha diversity analysis revealed that bioaugmentation with *B. velezensis* substantially altered the microbial community structure (Figure S2, Supplementary Materials). For the bacterial community, the Shannon index in the bioaugmented group was significantly higher than that in the control (CK) during the mid-to-late fermentation stages. This indicated that the introduction of the exogenous strain disrupted the dominance of a single genus (*Pediococcus*) observed in the CK group, establishing a dynamic balance co-dominated by *Bacillus* and *Pediococcus*, which significantly enhanced the bacterial community evenness. Conversely, both the Chao1 and Shannon indices of the fungal community were significantly lower in the bioaugmented group than in the CK group. This reduction was presumably attributed to the extracellular enzyme system of *B. velezensis*, which accelerated substrate saccharification and supplied abundant carbon and nitrogen sources for the rapid proliferation of *Saccharomyces*. This synergistically expedited the establishment of an absolute dominant niche for *Saccharomyces*, thereby exerting ecological exclusion on other low-abundance fungi within the system. These findings are consistent with those reported by Wang et al. [34].

**Figure 5:**
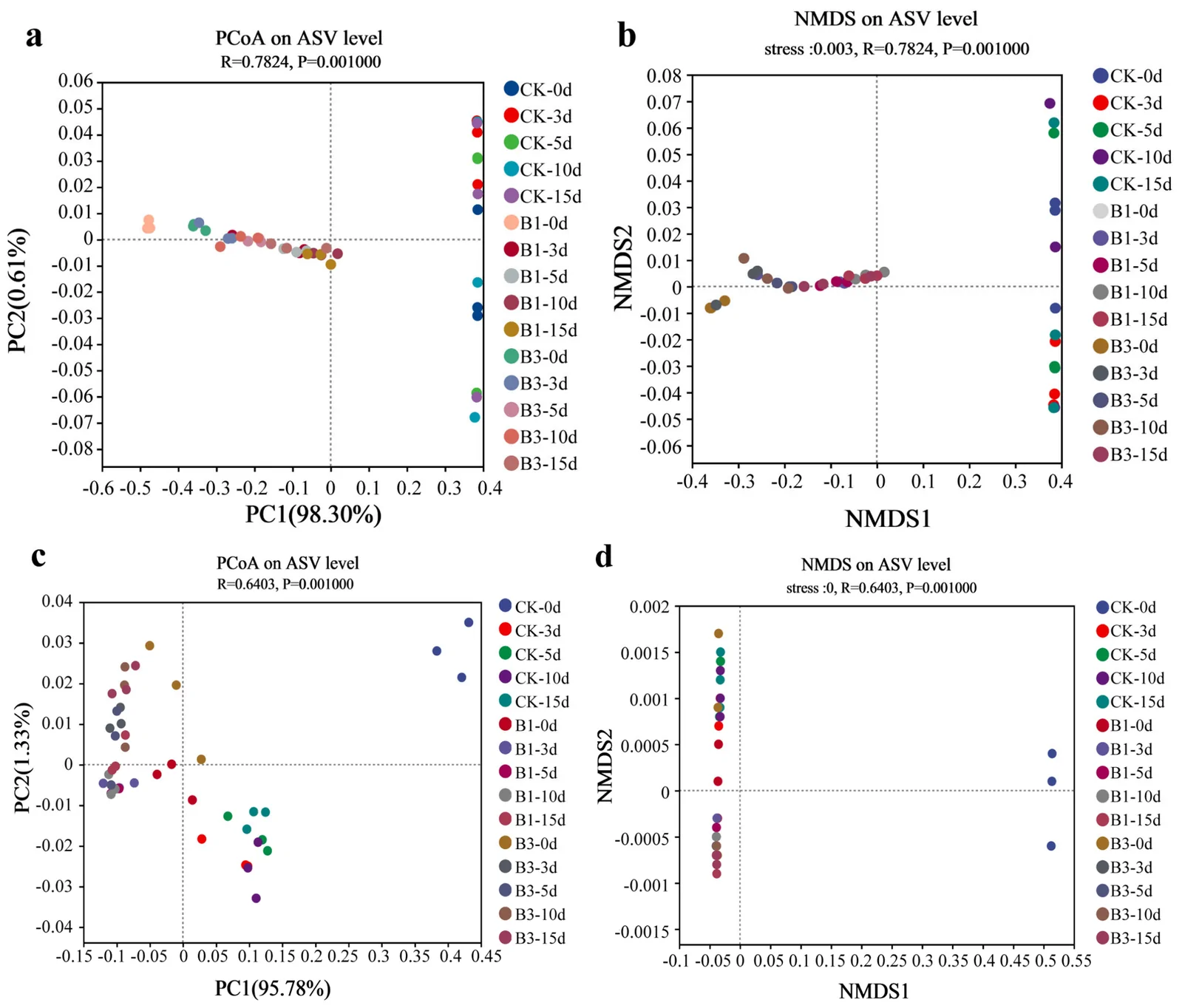
(**a**) Principal coordinate analysis (PCoA) of bacterial community; (**b**) non-metric multidimensional scaling (NMDS) analysis of bacterial community; (**c**) PCoA of fungal community; (**d**) NMDS analysis of fungal community during fermentation.

To clarify the dynamic changes in the microbial community during Xiaoqu Baijiu fermentation and the effects of inoculation treatments (B1, B3) on community structure, Principal Coordinates Analysis (PCoA) and Non-metric Multidimensional Scaling (NMDS) were employed to evaluate differences in microbial community structures (Figure 5). The results demonstrated significant structural differences in both bacterial and fungal communities between the bioaugmented groups (B1, B3) and the CK group (P = 0.001). In the PCoA analysis, the first principal coordinate (PCo1) accounted for 98.30% and 95.78% of the total variation in the bacterial and fungal communities, respectively, indicating that the addition of *B. velezensis* substantially reshaped the microbial composition. The stress values from the NMDS analysis were both below 0.01 (0.003 for bacteria and 0 for fungi), confirming the high reliability of the ordination results. Taken together, bioaugmentation with *B. velezensis* exerted distinct shaping effects on the bacterial and fungal communities: the bacterial community underwent more pronounced succession over fermentation time, whereas the fungal community remained relatively stable in terms of its overall dominant-genus framework, despite variations in abundance structure.

#### 3.2.2. Microbial Community Succession Across Fermentation Stages

##### Bacterial Community Succession Driven by *B. velezensis* Bioaugmentation

**Figure 6:**
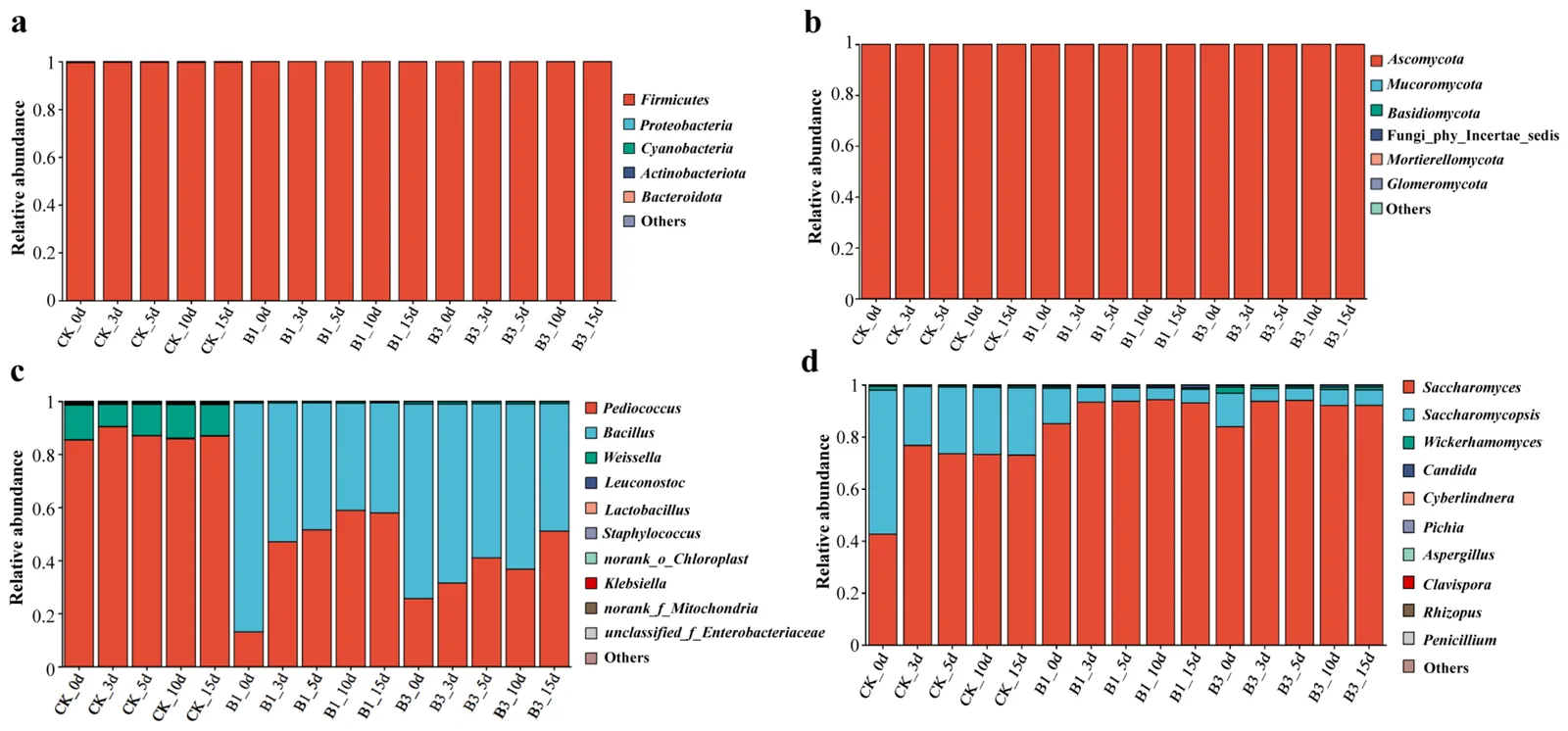
(**a**) Relative abundance of bacteria at the phylum level; (**b**) relative abundance of fungi at the phylum level; (**c**) relative abundance of bacteria at the genus level; (**d**) relative abundance of fungi at the genus level during the fermentation of Xiaoqu Baijiu.

Based on *16S rRNA gene* sequencing, 5 bacterial phyla were identified during Xiaoqu Baijiu fermentation. *Firmicutes* served as the absolute dominant phylum, maintaining a relative abundance >99% across all samples (Figure 6a). At the genus level (Figure 6c), *Pediococcus* (85.39–90.36%) and *Weissella* (8.37–12.98%) were the dominant bacterial genera in the CK group throughout fermentation, whereas *Bacillus* exhibited a low relative abundance (0.11–0.28%).

Inoculation with *B. velezensis* substantially restructured the bacterial community in *jiupei*. In the B1 group, the relative abundance of *Bacillus* reached 86.24% at the initial stage (day 0), subsequently undergoing a stage-wise decrease before stabilizing at 41.52% by the end of fermentation (day 15). Correspondingly, the relative abundance of *Pediococcus* increased from 13.03% to 57.90%, though remaining generally lower than that in the CK group; during late fermentation (days 10–15), *Pediococcus* and *Bacillus* reached a dynamic equilibrium. The ecological niche of *Weissella* was largely occupied by *Bacillus*, reducing its relative abundance at day 15 to only 0.44% (compared to 11.70% in CK group). Minor genera, including *Leuconostoc*, *Lactobacillus*, and *Staphylococcus*, also exhibited lower relative abundances than in the CK group. The B3 group displayed a bacterial community profile similar to B1, co-dominated by *Pediococcus* (25.60–51.06%) and *Bacillus* (48.04–73.40%), with genus-level temporal trends closely matching those of B1. These results demonstrate that *B. velezensis* bioaugmentation restructured the bacterial community by elevating *Bacillus* abundance to occupy a dominant ecological niche. This observation aligns with the findings reported by Song et al. [5], who demonstrated that inoculation with functional microorganisms (including *Bacillus*) reconstructed the community structure of Daqu.

##### Fungal Community Succession Driven by *B. velezensis* Bioaugmentation

At the fungal phylum level (Figure 6b), *Ascomycota* was the absolute dominant phylum across all groups (>99%). At the genus level (Figure 6d), *Saccharomyces* and *Saccharomycopsis* dominated the fungal community in the CK group throughout fermentation. As fermentation progressed in the CK group, the relative abundance of *Saccharomyces* rose from 42.56% to 73.00%, while *Saccharomycopsis* gradually decreased from 55.39% to 25.88%, reaching a dynamic equilibrium after day 5. The abundance of *Wickerhamomyces* (1.44% at day 0) displayed a decline-then-rise trajectory during fermentation; *Candida* exhibited a similar fluctuational trend.

Although *B. velezensis* bioaugmentation did not alter the core dominant fungal genera, it significantly altered their relative proportions. In the B1 group, the relative abundance of *Saccharomyces* reached 85.06% as early as day 0—exceeding the peak level of the CK group (76.72% on day 3)—and remained stable thereafter, indicating that bioaugmentation accelerated the establishment of *Saccharomyces* dominance. The temporal trend of *Saccharomycopsis* mirrored that of the CK group but remained consistently lower in overall abundance. The relative abundance of *Candida* exhibited an enrichment trend, increasing from 0.49% (day 0) to 1.07% (day 15), with potential contributions to the accumulation of esters and higher alcohols [35]. The B3 group shared similar dominant fungal genera and succession trends with B1, albeit with more pronounced community perturbations. *Wickerhamomyces* served as a dominant genus at day 0, dropped, and subsequently recovered to maintain a relatively high abundance at day 15.

In summary, *B. velezensis* bioaugmentation significantly reshaped the microbial community structure during Xiaoqu Baijiu fermentation. For bacteria, *B. velezensis* inoculation markedly increased *Bacillus* relative abundance, enabling it to dominate the early stage; as fermentation proceeded, *Pediococcus* recovered, and the two genera co-established a balanced dominant community during late fermentation. For fungi, bioaugmentation accelerated the enrichment of *Saccharomyces* while elevating the abundance of flavor-associated taxa such as *Candida*. Although B1 and B3 demonstrated high structural similarity, subtle differences in relative abundances and perturbation intensity highlight distinct strain-dependent capacities for inter-kingdom microbial regulation.

### 3.3. Correlation Analysis Between Microorganisms and Volatile Flavor Compounds

**Figure 7:**
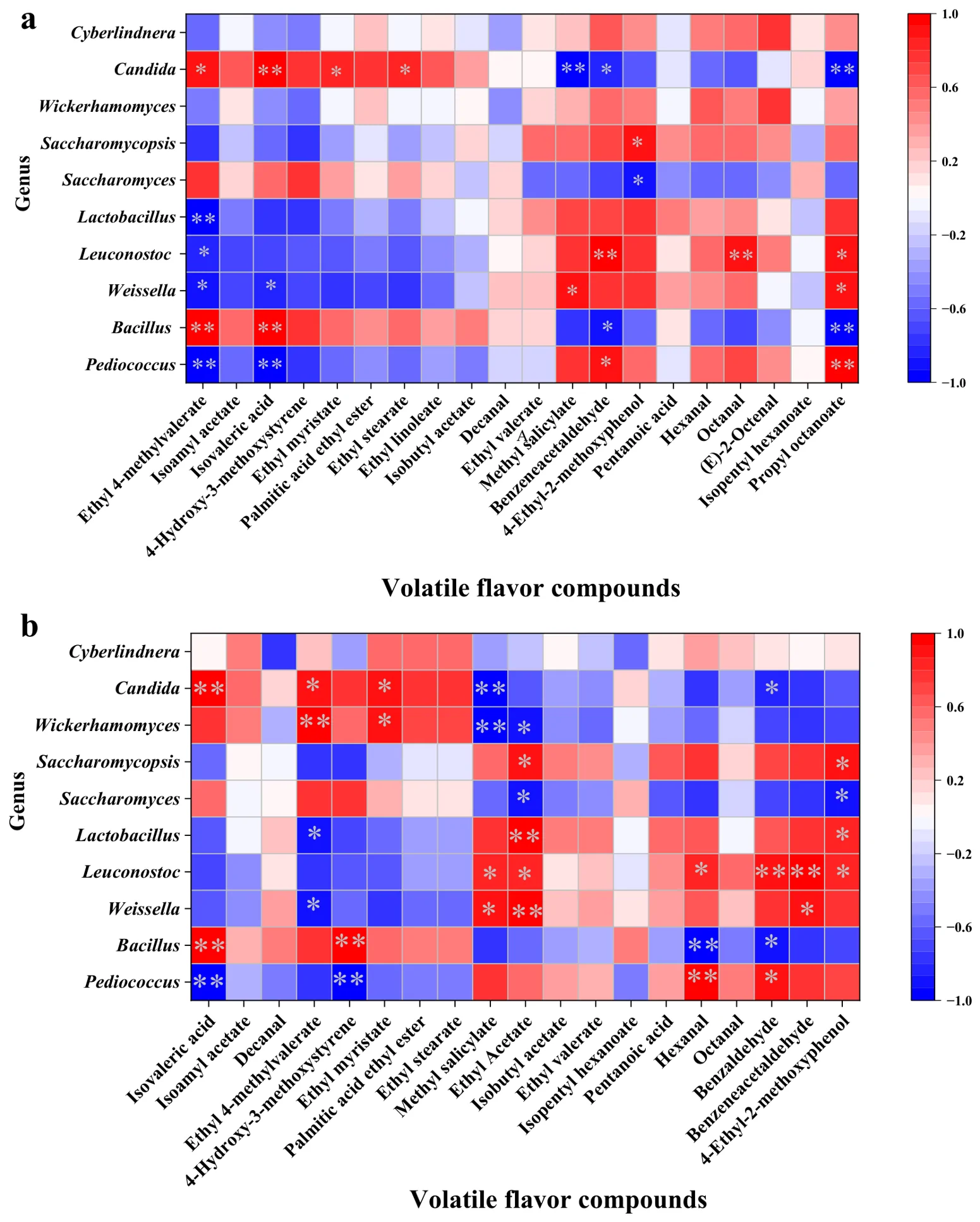
(**a**) Spearman correlation analysis between microorganisms and volatile flavor compounds in *jiupei* for CK and B1 groups; (**b**) Spearman correlation analysis between microorganisms and volatile flavor compounds in *jiupei* for CK and B3 groups. All correlations had *p* < 0.05 and |r| > 0.8, (*: *p* < 0.05, **: *p* < 0.01). The red color block represents the positive correlation between volatile components and microorganisms, and the blue color block represents the negative correlation between volatile components and microorganisms.

To elucidate the impact of microbial community succession on the formation of volatile metabolites during *B. velezensis* bioaugmentation, Spearman correlation analysis was conducted between major bacterial and fungal genera during late fermentation and differential volatile flavor compounds across groups (Figure 7).

In the comparative correlation analysis between the CK and B1 groups (Figure 7a), *Bacillus* exhibited significant associations with several key volatile flavor compounds. *Bacillus* showed a significant positive correlation with isovaleric acid and ethyl 4-methylvalerate (*p* < 0.01). It also displayed positive correlations with 2-methoxy-4-vinylphenol and LCFAEEs, including ethyl myristate, ethyl palmitate, and ethyl stearate, while exhibiting significant negative correlations with benzeneacetaldehyde and propyl octanoate (*p* < 0.05). These findings indicate that the introduction of exogenous *Bacillus* closely tracks with the accumulation of branched-chain flavor compounds and LCFAEEs. This effect is presumably attributable to the potential extracellular enzymatic capabilities of *B. velezensis*, which may enhance the enzymatic hydrolysis of substrates during fermentation to provide essential amino acid and fatty acid precursors for ester and phenol synthesis [36,37]. Furthermore, *Candida* was positively correlated with LCFAEEs such as ethyl myristate and ethyl palmitate (*p* < 0.05), indicating a positive statistical association with LCFAEE accumulation. Additionally, lactic acid bacteria (LAB, including *Lactobacillus*, *Leuconostoc*, *Weissella*, and *Pediococcus*) were positively correlated with methyl salicylate, benzeneacetaldehyde, 4-ethyl-2-methoxyphenol, and propyl octanoate, reflecting their statistical associations with the accumulation of aromatic aldehydes, volatile phenols, and short-to-medium-chain esters [38].

In the comparison between the CK and B3 groups (Figure 7b), the interactions between microorganisms and flavor metabolites revealed distinct regulatory patterns relative to the B1 group. *Bacillus* showed a significant positive correlation with isovaleric acid and 4-hydroxy-3-methoxystyrene (*p* < 0.01), positively correlated with esters such as ethyl 4-methylvalerate, ethyl myristate, ethyl palmitate, and ethyl stearate, and significantly negatively correlated with hexanal and benzaldehyde. This pattern indicates a positive statistical association between *Bacillus* relative abundance and long-chain ester levels and specific phenolic aromas in group B3, and may be associated with a decrease in the content of green aldehydes such as hexanal. Non-*Saccharomyces* yeasts, specifically *Candida* and *Wickerhamomyces*, exhibited positive correlations with multiple esters (including ethyl 4-methylvalerate, ethyl myristate, and ethyl palmitate), indicating that indigenous non-*Saccharomyces* populations co-align with the accumulation of long-chain esters and specific aroma precursors. In contrast, lactic acid bacteria (LAB; *Lactobacillus*, *Leuconostoc*, and *Weissella*) were positively correlated with aldehydes such as hexanal and octanal, but negatively correlated with the aforementioned fatty acid ethyl esters, a pattern that may relate to active LAB metabolism in connection with lipid degradation pathways and precursor aldehyde accumulation [39].

In summary, Spearman correlation analysis revealed distinct co-occurrence patterns between microbial taxa and volatile profiles. In the B1 group, the relative abundance of *Bacillus* was positively correlated with LCFAEEs and volatile phenols. In contrast, in the B3 group, *Bacillus* and indigenous non-*Saccharomyces* yeasts (*Candida* and *Wickerhamomyces*) covaried with ester accumulation, while the native LAB community was correlated with short-chain esters and aromatic aldehydes.

## 4. Discussion

Traditional Xiaoqu Baijiu fermentation predominantly employs semi-open systems, where the *Jiupei* micro-ecosystem relies heavily on starter-derived (Jiuqu) microorganisms. Substrate competition and spatial occupancy under these conditions often lead to the homogenization of metabolic networks [3]. In this study, bioaugmentation with exogenous *B. velezensis* strains B1 and B3 perturbed the initial micro-ecological equilibrium, accomplishing distinct regulatory impacts on both bacterial and fungal communities. At the bacterial level, *B. velezensis* bioaugmentation restructured the bacterial community succession pattern. Inoculation significantly elevated the relative abundance of *Bacillus*, rendering it the dominant taxon during early fermentation (peaking at 86.24%) and maintaining a high relative abundance exceeding 40% throughout the late stages. Together with *Pediococcus*, *Bacillus* formed a co-dominant niche, reflecting strong spatial occupancy and structural persistence within the fermentation matrix. Notably, *B. velezensis* is widely recognized in agricultural biocontrol for its capacity to synthesize lipopeptides, polyketides, and other secondary metabolites with broad-spectrum antimicrobial activities against diverse fungal and bacterial pathogens [40]. Within the current fermentation micro-ecosystem, however, *B. velezensis* introduction did not compromise the dominant status of *Saccharomyces cerevisiae*. Instead, it synchronized with the accelerated establishment of *S. cerevisiae* dominance, accompanied by an increased relative abundance of flavor-associated yeasts such as *Candida*.

Although strains B1 and B3 displayed broadly similar regulatory patterns regarding bacterial community succession, distinct strain-specific signatures emerged in their terminal volatile metabolite profiles. This divergence may stem from micro-scale differences in transcriptional regulation and extracellular enzymatic activities among strains. Even within the same species (*B. velezensis*), distinct isolates may vary in the expression efficiency of lipopeptide biosynthetic gene clusters, secretion rates of extracellular hydrolases, or physiological responses to indigenous metabolites. These variations could alter precursor supply rates or fine-tune micro-ecological interaction intensities, ultimately giving rise to distinct ester enrichment patterns. Prior studies have largely focused on using multi-strain consortia for flavor enhancement or employing *Bacillus* specifically to boost pyrazine accumulation [41,42,43]. In contrast, bioaugmentation with *B. velezensis* B1 and B3 in this study coincided with a substantial expansion in both the diversity and concentration of late-stage esters and alcohols. In particular, long-chain fatty acid ethyl esters (LCFAEEs)—such as ethyl laurate and ethyl arachidate—were markedly upregulated. This trend co-aligned with enhanced floral and fruity notes while offering a potential positive contribution to the tactile smoothness of the spirit [28]. These observations indicate that *B. velezensis* bioaugmentation may selectively facilitate specific ester synthesis pathways through a unique micro-ecological perturbation mode.

Based on these ecological and metabolic trends, we propose a possible metabolic synergy mechanism hypothesis: First, fungi such as *S. cerevisiae* may possess intrinsic physiological tolerance to the lipopeptides produced by *Bacillus* [44]. Concurrently, *B. velezensis* harbors the genetic capacity to secrete diverse extracellular enzymes (e.g., amylases, proteases, and cellulases). Exogenous inoculation may indirectly accelerate macromolecular substrate degradation, thereby supplying abundant carbon and nitrogen sources for yeast proliferation [45,46]. This metabolic support likely synchronizes with the rapid establishment of S. cerevisiae dominance and downstream flavor ester accumulation [47]. Second, exogenous perturbation reshaped niche partitioning among native dominant genera. The introduction of *B. velezensis* may have altered in situ carbon flux distribution and precursor availability, indirectly favoring core flavor metabolite accumulation. This hypothesis nevertheless requires further validation through metabolomic and flux analysis.

Finally, several inherent limitations of this study warrant consideration. First, because extracellular enzymatic activities, amino acid and fatty acid precursor metabolic pools, metabolic fluxes, and absolute microbial abundances were not directly quantified, the proposed “substrate degradation–precursor accumulation–yeast synergy” pathway remains a biochemically plausible hypothesis rather than an empirically established mechanism. Second, high-throughput amplicon sequencing inherently reflects relative community abundances rather than absolute cell counts, and statistical co-occurrence network analyses cannot directly prove definitive causal relationships between microbial succession and flavor metabolite dynamics. Furthermore, the fermentations in this study were conducted at a laboratory scale, where micro-ecological dynamics—such as oxygen gradients, heat dissipation, and mass transfer kinetics—may diverge from those operating in large-scale industrial Baijiu production. Additionally, flavor profiling was restricted to the fermented matrix (*jiupei*), without tracking volatile migration behavior into the final distillate or evaluating the sensory properties of the distilled spirit itself. Therefore, prospective studies are required to evaluate and validate the bioaugmentation performance of *B. velezensis* under pilot-scale and industrial manufacturing conditions. Integrating quantitative amplicon sequencing, multi-omics metabolic flux analysis, and distillation trial profiling will be essential to fully decipher the complete mechanism by which *B. velezensis* dictates quality formation in Xiaoqu Baijiu.

## 5. Conclusions

This study demonstrates that bioaugmentation with exogenous *B. velezensis* strains B1 and B3 significantly alters both the micro-ecological architecture and the volatile metabolic dynamics during Xiaoqu Baijiu fermentation. Inoculation with *B. velezensis* exerted distinct regulatory effects on the indigenous microbiome: at the bacterial level, it substantially restructured community succession, establishing a co-dominant ecological niche between *Bacillus* and *Pediococcus* during the middle-to-late fermentation stages; at the fungal level, this transition co-occurred with an elevated relative abundance of *Saccharomyces cerevisiae* and key functional yeasts. These micro-ecological shifts closely aligned with volatile metabolite enrichment. Bioaugmented fermentation markedly enhanced the accumulation of LCFAEEs—including ethyl laurate, ethyl myristate, and ethyl palmitate—which offer a positive potential contribution to floral/fruity notes and tactile smoothness. Spearman co-occurrence analysis further revealed that *Bacillus* positively aligned with core esters, including ethyl 4-methylvalerate, ethyl myristate, ethyl palmitate, and ethyl stearate. Overall, these findings highlight a promising strategy for employing *B. velezensis* to modulate micro-ecological dynamics and shape the flavor profile of Xiaoqu Baijiu, offering a rational theoretical foundation for targeted micro-ecological regulation and quality optimization in traditional semi-solid distilled spirits.

## Data Availability

The original contributions presented in this study are included in the article/Supplementary Material. Further inquiries can be directed to the corresponding authors.

## Supplementary Materials

The following supporting information can be downloaded at: https://www.mdpi.com/article/10.3390/foods15203714/s1, Figure S1: Production process of Xiaoqu Baijiu. Figure S2: (**a**) Chao1 index of bacteria; (**b**) Shannon index of bacteria; (**c**) Chao1 index of fungi; (**d**) Shannon index of fungi during *jiupei* fermentation of Xiaoqu Baijiu. Chao1 and Shannon indices were compared using Kruskal–Wallis test followed by Dunn’s multiple-comparison test (non-parametric). Figure S3: Changes in volatile flavor compounds in *jiupei* during fermentation. (**a**) CK; (**b**) B1; (**c**) B3. Table S1: Types and contents of flavor substances in each group during fermentation. Table S2: Content of volatile flavor compounds and OAV.
