# Supplementary material for: Differential Regulation of Microbial Community Succession by Bacillus velezensis and Its Impact on Flavor Compound Synthesis During Xiaoqu Baijiu Fermentation Stage
Source: Foods. 2026 Oct 19;15(20):3714. doi: 10.3390/foods15203714 (PMC13704129; doi:10.3390/foods15203714)
Supplement: Supplementary file 1 [file foods-15-03714-s001.zip › Supplementary Material.pdf]

Supplementary Material

1 Figures

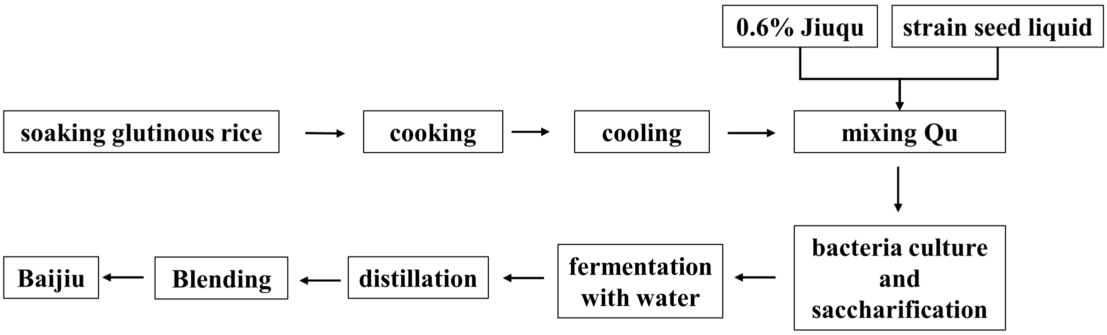

Figure S1. Production process of Xiaoqu Baijiu

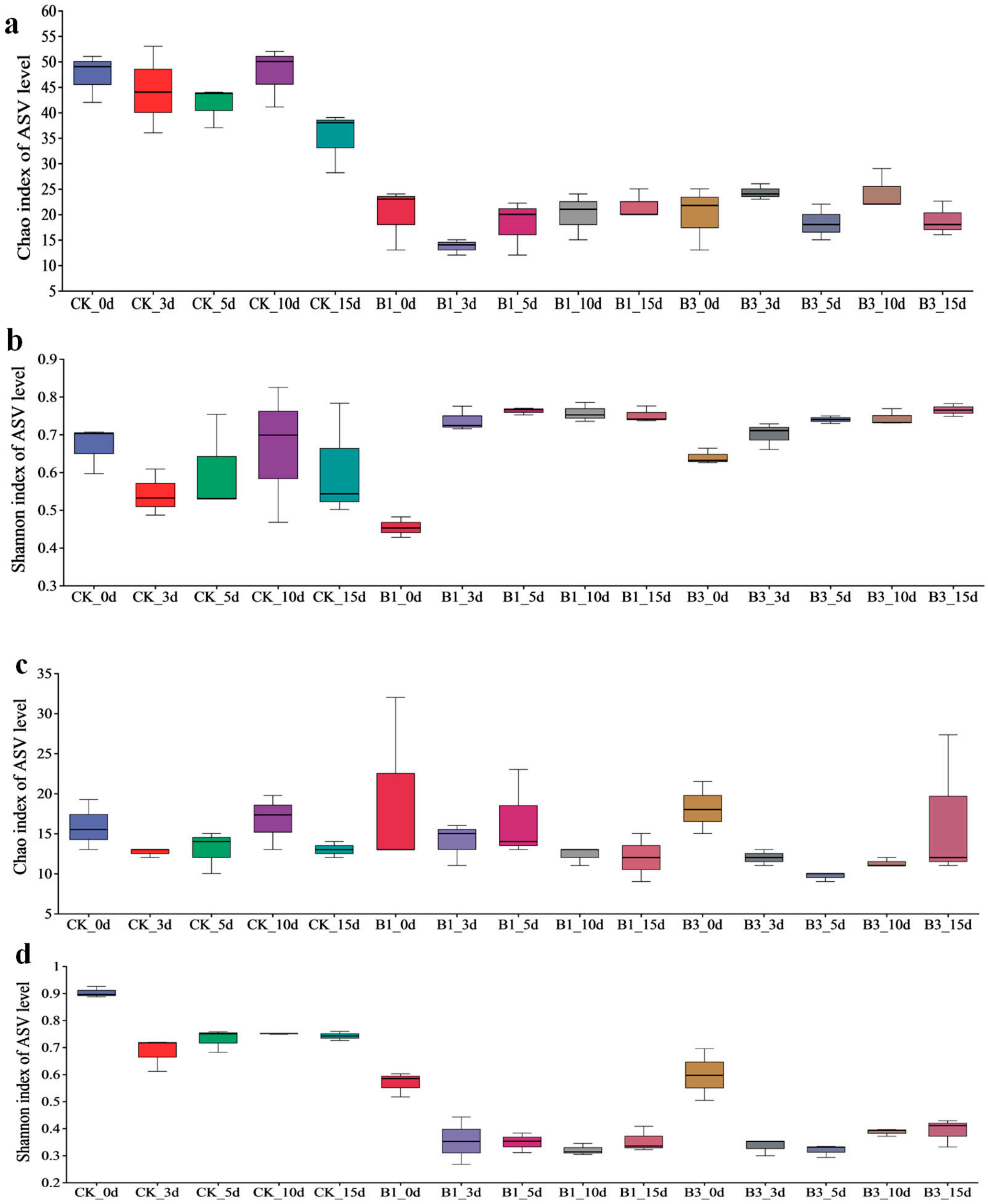

Figure S2. (**a**) Chao1 index of bacteria; (**b**) Shannon index of bacteria; (**c**) Chao1 index of fungi; (**d**) Shannon index of fungi during *jiupei* fermentation of Xiaoqu Baijiu. Chao1 and Shannon indices were compared using Kruskal－Wallis test followed by Dunn's multiple-comparison test (non-parametric).

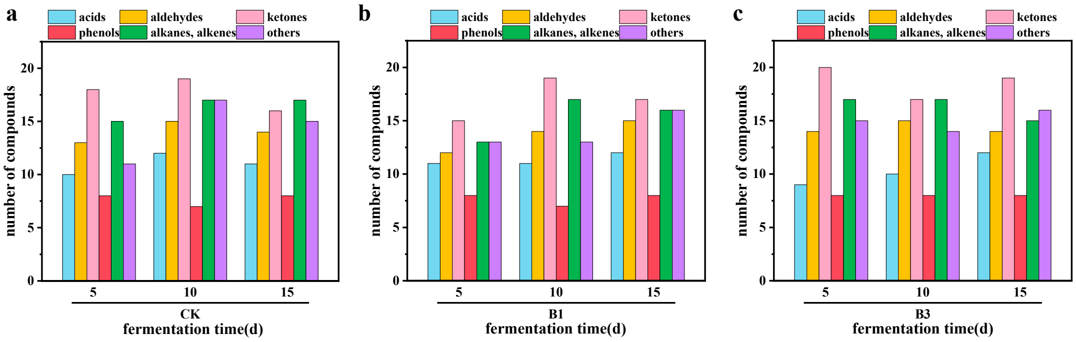

Figure S3. Changes in volatile flavor compounds in *jiupei* during fermentation. **(a)** CK; **(b)** B1; **(c)** B3

Table S1. Types and contents of flavor substances in each group during fermentation

group		number of species / compound content (mg/L)								
		CK-5d	CK-10d	CK-15d	B1-5d	B1-10d	B1-15d	B3-5d	B3-10d	B3-15d
esters	number of species	65	76	80	75	79	82	82	82	85
	content	4046.140	5770.162	6068.055	5916.408	5949.869	7087.494	5558.779	6562.386	6774.702
alcohols	number of species	41	43	40	40	41	43	41	42	40
	content	5469.398	6392.343	6172.914	6858.329	6032.172	6644.748	5819.228	6012.802	6488.862
acids	number of species	10	12	11	11	11	12	9	10	12
	content	205.949	282.207	247.533	262.368	257.336	328.209	193.884	256.891	248.728
aldehydes	number of species	13	15	14	12	14	15	14	15	14
	content	102.048	77.904	69.154	57.575	60.868	59.533	51.158	60.270	61.667
ketones	number of species	18	19	16	15	19	17	20	17	19
	content	166.096	188.297	170.585	142.215	159.596	175.421	172.809	182.067	184.094
phenols	number of species	8	7	8	8	7	8	8	8	8
	content	950.428	1215.522	4487.955	1321.972	83.586	1170.924	1004.446	1475.746	4720.986
alkanes and alkenes	number of species	15	17	17	13	17	16	17	17	15
	content	635.135	263.437	142.799	100.272	195.279	144.465	126.996	100.771	94.696

Continued Table S1 Types and contents of flavor substances in each group during fermentation

group		number of species / compound content (mg/L)								
		CK-5d	CK-10d	CK-15d	B1-5d	B1-10d	B1-15d	B3-5d	B3-10d	B3-15d
others	number of species	11	17	15	13	13	16	15	14	16
	content	79.500	109.433	92.895	100.533	134.175	136.543	103.578	116.725	115.276
total number of species		180	205	200	186	200	208	205	204	208
total content		11653.75	14298.36	17450.94	14759.07	12872.14	15746.53	13030.13	14766.98	18688.29
